# Cellular glutathione as a determinant of the sensitivity of colorectal tumour cell-lines to ZD2767 antibody-directed enzyme prodrug therapy (ADEPT)

**DOI:** 10.1054/bjoc.2000.1240

**Published:** 2000-06-15

**Authors:** N R Monks, J A Calvete, N J Curtin, D C Blakey, S J East, D R Newell

**Affiliations:** Cancer Research Unit, University of Newcastle upon Tyne, Tyne and Wear, NE2 4HH, UK; Cancer and Infection Bioscience Department, AstraZeneca Pharmaceuticals, Alderley Park, Macclesfield, Cheshire, SK10 4TG, UK

**Keywords:** glutathione, ZD2767, ADEPT, alkylating agent, buthionine sulfoximine

## Abstract

ZD2767P, a nitrogen mustard glutamate prodrug, is currently being evaluated in Phase 1 clinical trials of antibody directed enzyme prodrug therapy (ADEPT). There was no significant relationship between basal glutathione (GSH) concentration and sensitivity to ZD2767P + carboxpeptidase G2 (CPG2) in colorectal tumour cell-lines. Depletion of intracellular GSH using buthionine sulfoximine (BSO) resulted in only a modest potentiation of ZD2767P + CPG2 activity and hence BSO is unlikely to markedly enhance the activity of this ADEPT treatment. © 2000 Cancer Research Campaign


British Journal of Cancer (2000) 83(2), 267–269
© 2000 Cancer Research Campaign
doi: 10.1054/ bjoc.2000.1240, available online at http://www.idealibrary.com on IDEAL®

### Cellular glutathione as a determinant of the sensitivity of colorectal tumour cell-lines to ZD2767 antibody-directed enzyme prodrug therapy (ADEPT)

**NR Monks[1], JA Calvete[1], NJ Curtin, DC Blakey[2], SJ East[2] and DR Newell[1]**

[1]Cancer Research Unit, University of Newcastle upon Tyne, Tyne and Wear, NE2 4HH, UK; [2]Cancer and Infection Bioscience Department, AstraZeneca Pharmaceuticals, Alderley Park, Macclesfield, Cheshire, SK10 4TG, UK

**Summary** ZD2767P, a nitrogen mustard glutamate prodrug, is currently being evaluated in Phase 1 clinical trials of antibody directed enzyme prodrug therapy (ADEPT). There was no significant relationship between basal glutathione (GSH) concentration and sensitivity to ZD2767P + carboxpeptidase G2 (CPG2) in colorectal tumour cell-lines. Depletion of intracellular GSH using buthionine sulfoximine (BSO) resulted in only a modest potentiation of ZD2767P + CPG2 activity and hence BSO is unlikely to markedly enhance the activity of this ADEPT treatment. © 2000 Cancer Research Campaign

**Keywords:** glutathione; ZD2767; ADEPT; alkylating agent; buthionine sulfoximine

ZD2767 is the latest antibody directed enzyme prodrug therapy (ADEPT) system to be developed and is currently undergoing Phase 1 clinical evaluation in patients with carcinoembryonic antigen (CEA)-expressing tumours. The ZD2767 ADEPT system utilizes the A5B7 $F(ab')_2$-carboxypeptidase G2 (CPG2) conjugate (ZD2767C) which is targeted at CEA-expressing tumours. The enzyme specifically activates a di-iodophenol mustard glutamate prodrug (4-[N,N-bis(2-iodoethyl)amino]phenoxycarbonyl L-glutamic acid, ZD2767P, Figure 1A) to the potent di-iodophenol mustard (4-[N,N-bis(2-iodoethyl)amino]phenol, ZD2767D, Figure 1B), via the cleavage of the carbamate bond releasing the glutamate moiety (Springer et al, 1995; Blakey et al, 1996).

Previously, it has been shown that ZD2767P + CPG2 is a potent cytotoxic treatment, which produces concentration-dependent DNA–DNA inter-strand cross-links as the primary cytotoxic lesion (Monks et al, unpublished observations). Maximum levels of cross-links are formed within 10 min of drug exposure, and levels of cross-links determine, in part, the cytotoxicity of ZD2767P + CPG2. However, the extracellular ZD2767P + CPG2 concentration required to induce similar levels of DNA cross-linking and cytotoxicity varied 10-fold in a panel of colorectal and non-small-cell lung cancer cell-lines, suggesting that cellular factors prior to DNA interaction determine the levels of cross-linking achieved for a given extracellular concentration.

Numerous cellular mechanisms have been identified which can determine the activity of nitrogen mustards. These include drug influx and efflux, decreased drug activation, cellular detoxification (chemical and enzymatic), repair of drug-induced lesions and a reduced ability to initiate apoptosis (Hayes and Wolf, 1990; Hall and Tilby, 1992). Of the potential determinants of ZD2767 cytotoxicity, reduced influx, enhanced efflux or impaired activation are unlikely to be important, because ZD2767D is a low molecular weight uncharged direct-acting alkylating agent. As indicated above, levels of ZD2767-induced DNA–DNA cross-linking determine, in part, cytotoxicity. Therefore, variation in the cellular detoxification of ZD2767D prior to reaction with DNA, specifically by reaction with glutathione (GSH), may explain differences between cell-lines in their sensitivity to ZD2767P + CPG2.

The aim of the studies reported here were to investigate the relationship between basal GSH concentration and the sensitivity of

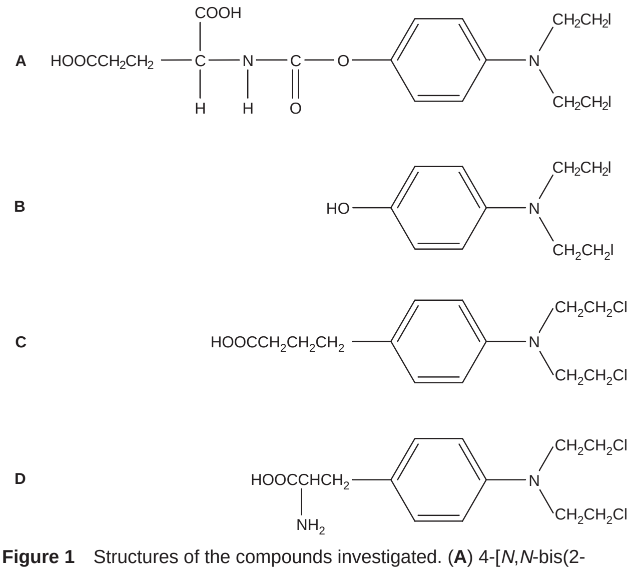

**Figure 1** Structures of the compounds investigated. (**A**) 4-[*N*,*N*-bis(2-iodoethyl)amino]phenoxycarbonyl L-glutamic acid prodrug (ZD2767P), (**B**) 4-[*N*,*N*-bis(2-iodoethyl)amino]phenol drug (ZD2767D), (**C**) chlorambucil and (**D**) melphalan.

*Received 21 December 1999*
*Revised 2 March 2000*
*Accepted 10 March 2000*

*Correspondence to:* DR Newell colorectal cell-lines to ZD2767P + CPG2 and, for comparison, chlorambucil (Figure 1C). Buthionine sulfoximine (BSO)-mediated depletion of GSH was also investigated to establish the extent of the potentiation of ZD2767P + CPG2 activity that could be achieved compared to the classical nitrogen mustards chlorambucil and melphalan (Figure 1D).

### MATERIALS AND METHODS

The prodrug ZD2767P was synthesized as described previously by Springer et al (1995). Chlorambucil and melphalan were purchased from Sigma (Poole, Dorset, UK). CPG2 was provided by CAMR (Salisbury, Wiltshire, UK). The colorectal tumour cell-lines HT29, LoVo, LS174T, HCT116, HCT15 and SW620 were obtained from the European Collection of Animal Cell Cultures (ECACC, Wiltshire, UK). All cell-lines were maintained in RPMI 1640 (complete) medium containing 2 mM L-glutamine (Gibco-BRL Life Technologies Ltd, Paisley, UK), supplemented with 10% (v/v) foetal calf serum (Globepharm Ltd, Esher, Surrey, UK), and incubated at 37°C in 5% $CO_2$. All cell-lines were routinely tested for mycoplasma using the method of Chen (1977). All other reagents were supplied by Sigma Chemical Co (Poole, Dorset, UK), unless stated otherwise.

### Growth inhibition studies

The tumour cell-lines were incubated with ZD2767P + CPG2 (0.1 unit well$^{-1}$), chlorambucil or melphalan at the indicated concentrations, in 96-well microtiter plates ($1 \times 10^3$–$1 \times 10^4$ cells well$^{-1}$) for 1 h. The medium was subsequently removed and the cells incubated in 200 µl of drug-free medium for a further 96 h. Cell growth was determined using the sulphorhodamine B (SRB) dye assay as previously described by Skehan et al (1990).

### Glutathione determination

Intracellular GSH levels were determined by HPLC using adaptations of previous methods (Cotgreave and Moldeus, 1986; Hogarth et al; 1999). Cell volumes were determined using a Coulter counter (Model Z1, Coulter Electronics, UK).

### RESULTS

GSH concentrations in each of the cell-lines were determined in duplicate in each experiment and expressed as the absolute mM cellular GSH concentration. GSH concentrations varied between the cell-lines (Table 1) with HT29 and LS174T cells having the highest basal GSH concentrations (5.5 and 4.6 mM, respectively), and HCT15 cells the lowest (0.9 mM).

To investigate the possible role of GSH as a determinant of the in vitro sensitivity of colorectal cell-lines to ZD2767P + CPG2, the levels of GSH were compared with the $IC_{50}$ data generated for ZD2767P + CPG2 and chlorambucil (Table 2). No clear relationship was identified between GSH concentration and sensitivity to either ZD2767P + CPG2 or chlorambucil (linear regression analysis, $r^2 < 0.05$, $P > 0.67$).

To determine the effect of GSH depletion in individual cell-lines on sensitivity to ZD2767P + CPG2, HT29, LoVo and LS174T cells were treated with 10 µM BSO for 24 h, a non-toxic concentration which reduced the levels of total intracellular GSH by 73–83% (Table 3). BSO pre-treatment was shown to sensitise all 3 cell lines to ZD2767P + CPG2 (Table 3). However, the effect was not statistically significant in either the HT29 or LS174T cell-lines, with 10 µM BSO producing only a 2.3 and 1.9-fold reduction in $IC_{50}$ values, respectively.

To assess whether the level of BSO-mediated potentiation seen with ZD2767P + CPG2 was comparable to that achievable with classical nitrogen mustards, HT29 cells were exposed to 10 µM BSO for 24 h prior to a 1 h exposure to ZD2767P + CPG2, chlorambucil or melphalan. GSH depletion by 10 µM BSO was shown to increase the sensitivity of HT29 cells to all three alkylating agent treatments by 2.2–3-fold (Table 4). Although an increase in

**Table 1** Basal GSH levels in human colorectal tumour cell-lines

| Cell-line | Cell volume (picolitres) | Intracellular GSH concentration (mM) |
|---|---|---|
| HT29 | 2.24 ± 0.08 | 5.5 ± 0.89 |
| LS174T | 1.32 ± 0.04 | 4.6 ± 0.20 |
| HCT116 | 1.52 ± 0.13 | 2.8 ± 0.66 |
| LoVo | 1.46 ± 0.12 | 2.2 ± 0.34 |
| SW620 | 1.32 ± 0.04 | 2.2 ± 0.38 |
| HCT15 | 1.39 ± 0.11 | 0.9 ± 0.07 |

Results were generated by gradient HPLC analysis as described in the methods. Values are the mean ± S.D of ≥ 3 experiments

**Table 2** $IC_{50}$ values for growth inhibition in colorectal cell-lines treated with ZD2767P + CPG2 or chlorambucil

| Cell-line | ZD2767P + $CPG_2$ $IC_{50}$ (µM) | Chlorambucil $IC_{50}$ (µM) |
|---|---|---|
| HCT116 | 1.1 ± 0.47 | 277 ± 106 |
| HT29 | 0.82 ± 0.62 | 201 ± 61 |
| HCT15 | 0.78 ± 0.07 | 76 ± 11 |
| LoVo | 0.41 ± 0.14 | 79 ± 5 |
| SW620 | 0.25 ± 0.01 | 66 ± 5 |
| LS174T | 0.04 ± 0.03 | 26 ± 4 |

Cells were exposed to ZD2767P + 1 unit ml$^{-1}$ CPG2 or chlorambucil for 1 h, followed by 96 h incubation in drug-free medium. Growth inhibition was assessed using the SRB assay. Values are the mean ± S.D of ≥ 3 experiments

**Table 3** Depletion of GSH and the potentiation of ZD2767P + CPG2-induced growth inhibition in colorectal tumour cell-lines following a 24 h exposure to 10 µM BSO

| Cell-line | BSO concentration (µM) | GSH content (% Control) | ZD2767P + $CPG_2$ $IC_{50}$ (µM) | Reduction in $IC_{50}$ (fold) |
|---|---|---|---|---|
| HT29 | 0 | | 0.73 ± 0.2 | |
| | 10 | 17 | 0.32 ± 0.04 (ns) | 2.3 |
| LoVo | 0 | | 0.38 ± 0.08 | |
| | 10 | 27 | 0.19 ± 0.03 ($P < 0.05$) | 2 |
| LS174T | 0 | | 0.021 ± 0.003 | |
| | 10 | 25 | 0.011 ± 0.008 (ns) | 1.9 |

GSH levels were determined using gradient HPLC analysis following 24 h exposure to BSO, and growth inhibition following 1 h exposure to ZD2767P + 1 unit ml$^{-1}$ CPG2 using the SRB dye assay after 96 h drug-free incubation. The data given are the mean ± S.D for three separate experiments. Statistical analysis was performed using the two-tailed unpaired Welch's *t*-test

 © 2000 Cancer Research Campaign

**Table 4** $IC_{50}$ values for the growth inhibition of HT29 colorectal tumour cells after 1 h exposure to ZD2767P + CPG2, chlorambucil or melphalan following 24 h exposure with 10 μM BSO

| Drug | $IC_{50}$ (μM) | $IC_{50}$ (μM) + 10 μM BSO | Reduction in $IC_{50}$ (fold) |
|---|---|---|---|
| ZD2767P + CPG2 | 0.47 ± 0.31 | 0.16 ± 0.12 | 3 ± 0.9 |
| Chlorambucil | 270 ± 120 | 120 ± 54 | 2.2 ± 0.4 |
| Melphalan | 63 ± 26 | 29 ± 19 | 2.5 ± 0.7 |

Growth inhibition was assessed using the SRB dye assay after 96 h incubation in drug-free medium. The data are the mean ± S.D. of three separate experiments. Statistical analysis was performed using the two-tailed paired Students *t*-test sensitivity was observed, statistical analyses (paired students *t*-test) showed that the effect was only significant at the 5% level in the case of melphalan ($P$ = 0.02), but not for ZD2767P + CPG2 or chlorambucil ($P$ = 0.13 and 0.07, respectively).

### DISCUSSION

The studies reported in this paper were performed to determine the role of intracellular GSH as a determinant of the sensitivity of colorectal cell-lines to ZD2767P + CPG2. Analysis of basal GSH levels identified differences between the colorectal tumour cell-lines, concentrations ranging from 5.5 mM in HT29 cells to as low as 0.9 mM in HCT15 cells. The lack of any clear relationship between sensitivity to ZD2767P + CPG2 or chlorambucil and basal GSH levels suggests that other cellular factors are more important in determining inherent sensitivity in the cell-lines studied. GSH concentration has previously been shown to correlate with nitrogen mustard sensitivity in resistant tumour cell-lines, which suggests that acquired resistance but not inherent sensitivity to nitrogen mustards can be related, in part, to intracellular GSH content (Alaoui-Jamali et al, 1992; Friedman et al, 1994).

To investigate further the potential role of GSH as a determinant of the sensitivity of colorectal tumour cells to ZD2767P + CPG2 and chlorambucil, buthionine sulfoximine (BSO) was used to inhibit GSH synthesis and deplete GSH-levels prior to drug treatment. Although GSH depletion sensitized all three cell-lines to ZD2767P + CPG2, the effects were modest and, with the exception of the LoVo cell-line, not significant at the 5% level.

To compare the effects of GSH depletion on the potentiation of ZD2767P + CPG2, chlorambucil or melphalan activity, HT29 colorectal tumour cells were pre-treated with 10 μM BSO. This non-toxic BSO concentration produced an 83% depletion of GSH levels but only a modest potentiation of the activity of the three agents, (only significant in the case of melphalan).

There are a number of factors which could explain the lack of any marked (i.e. > 3-fold) potentiation following BSO-mediated depletion of GSH, both in this study and in similar studies with nitrogen mustards reported previously. A major consideration is that despite achieving > 73% depletion of intracellular GSH, residual GSH concentrations are still in the mM range, i.e. far in excess of the extracellular drug concentrations used in these investigations. Furthermore, GSH compartmentalization, specifically in the nucleus, may explain the lack of potentiation seen following BSO-mediated GSH depletion (Smith et al, 1996).

In conclusion, these studies have demonstrated that cellular sensitivity to the ADEPT components ZD2767P + CPG2 is not related to intracellular GSH levels. BSO-mediated GSH depletion by as much as 83% resulted in only a modest potentiation of ZD2767P + CPG2 activity (2–3 fold), a magnitude similar to that observed with the classical nitrogen mustards chlorambucil and melphalan. On the basis of these data, tumour GSH levels are unlikely to be a determinant of the clinical activity of ZD2767 ADEPT, and combination studies involving BSO are not indicated.

### ACKNOWLEDGEMENTS

The authors are grateful to Mr Gordon Taylor for his assistance in developing the GSH HPLC assay, and Drs Andrew Hall and Linda Hogarth for their advice. This study was supported by grants from the Cancer Research Campaign and AstraZeneca Pharmaceuticals.

### REFERENCES

Alaoui-Jamali MA, Panasci L, Centurioni GM, Schecter R, Lehnert S and Batist G (1992) Nitrogen mustard-DNA interaction in melphalan-resistant mammary carcinoma cells with elevated intracellular glutathione and glutathione-*S*-transferase activity. *Cancer Chemother Pharmacol* **30**: 341–347

Blakey DC, Burke PJ, Davies DH, Dowell RI, East SJ, Eckersley KP, Fitton JE, McDaid J, Melton RG, Niculescu-Duvaz IA, Pinder PE, Sharma SK, Wright AF and Springer CJ (1996) ZD2767, an improved system for antibody-directed enzyme prodrug therapy that results in tumour regressions in colorectal tumour xenografts. *Cancer Res* **56**: 3287–3292

Chen T (1977) In situ detection of mycoplasma contamination in cell cultures by fluorescent Hoechst 33258 stain. *Exp Cell Res* **104**: 255–262

Cotgreave IA and Moldeus P (1986) Methodologies for the application of monobromobimane to the simultaneous analysis of soluble and protein thiol components of biological systems. *J Biochem Biophys Methods* **13**: 231–249

Friedman HS, Dolan ME, Kaufmann SH, Colvin OM, Griffith OW, Moschel RC, Schold SC, Bigner DD and Ali-Osman F (1994) Elevated DNA polymerase α, DNA polymerase β, and DNA topoisommerase II in a melphalan-resistant rhabdomyosarcoma xenograft that is cross resistant to nitrosoureas and topotecan. *Cancer Res* **54**: 3487–3493

Hall AG and Tilby MJ (1992) Mechanisms of action of, and modes of resistance to, alkylating agents used in the treatment of haematological malignancies. *Blood Rev* **6**: 163–173

Hayes JD and Wolf CR (1990) Molecular mechanisms of drug resistance. *Biochem J* **272**: 281–295

Hogarth LA, Rabello CMA and Hall AG (1999) Measurement of reduced glutathione using high-pressure liquid chromatography. *Methods in Molecular Medicine* **28**: 91–94

Skehan P, Storeng R, Scudiero D, Monks A, McMahon J, Vistica D, Warren JT, Bokesch H, Kenney S and Boyd MR (1990) New colorimetric cytotoxicity assay for anticancer-drug screening. *J Natl Cancer Inst* **82**: 1107–1112

Smith CV, Jones DP, Guenthner TM, Lash LH and Lauterburg BH (1996) Compartmentation of glutathione: implications for the study of toxicity and disease. *Toxicol Appl Pharmacol* **140**: 1–12

Springer CJ, Dowell R, Burke PJ, Hadley E, Davies DH, Blakey DC, Melton RG and Niculescu-Duvaz I (1995) Optimization of alkylating agent prodrugs derived from phenol and aniline mustards: a new clinical candidate prodrug (ZD2767) for antibody-directed enzyme prodrug therapy (ADEPT). *J Med Chem* **38**: 5051–5065

*© 2000 Cancer Research Campaign*
